# Selective activity of Oleanolic and Maslinic Acids on the Amastigote form of *Leishmania* Spp

**Published:** 2017

**Authors:** Ines Sifaoui, Atteneri López-Arencibia, Carmen M Martín-Navarro, María Reyes-Batlle, Mondher Mejri, Basilio Valladares, Jacob Lorenzo-Morales, Manef Abderabba, José Enrique Piñero

**Affiliations:** a *Laboratoire Matériaux-Molécules et Applications, IPEST, B.P 51 2070, La Marsa, University of Carthage, Tunisia. *; b *University Institute of Tropical Diseases and Public Health, University of La Laguna, Avda Francisco Sanchez s/n, Campus de Anchieta, 38271 La Laguna Tenerife, Canary Islands, Spain*.

**Keywords:** Oleanolic acid, Maslinic acid, Triterpenic acids, Anti-amastigotes activity, Selectivity index

## Abstract

Leishmaniasis represents a serious threat to the health as one of the most important neglected tropical diseases as designated by the World Health Organization. The disease is endemic in 82 countries, among them Tunisia is an indigenous area for cutaneous Leishmaniasis. In a previous work, two tritepenic acids namely oleanolic and maslinic acids have been isolated from olive leaf extract. In the present paper, the *in vitro* activity against amastigotes stage of Leishmania (L.) infantum and Leishmania (L.) *amazonensis *was investigated. Maslinic acid showed the highest activity, against *L. amazonensis, *with an IC_50_ of 1.417 ± 0.401 µg/mL and a selectivity index of 9.405. Although, the oleanolic acid exhibit a better activity against *L. infantum *with an IC_50_ of 0.999 ± 0.089 µg/mL and selectivity index of 8.111.

## Introduction

Leishmaniasis represents a serious threat to the health as one of the most important neglected tropical diseases as designated by the World Health Organization (WHO) (1). Accordingly, about 2 million new cases occur every year and over 12 million people are presently infected (2) 

Parasite from the genus Leishmania present a complex life cycle that involves both vertebrate and invertebrate hosts and two developmental stages: promastigotes, the proliferative form found in invertebrate host, and amastigotes, the proliferative form found inside the vertebrate host (3). 

The classic treatments of this disease have been compromised by the drugs toxicity and the appearance of new resistance forms. 

Therefore, the developing of new treatments strategy has been increased significantly. Natural products seem to be a good solution for those kinds of parasitosis. We have shown in previous study that triterpenic acids isolated form olive leaf extract namely oleanolic and maslinic acids, display inhibitory effects against the promastigotes form of *Leishmania amazonensis *and* infantum *(4). 

The aim of the present work is to study the selective activity of these compounds against the amasitogotes form of *Leishmania amazonensis *and *Leishmania infantum*. 

## Experimental

Both tritepenic acids, oleanolic and maslinic, have been isolated from olive leaf extract in a previous study (4). 

**Figure 1:**
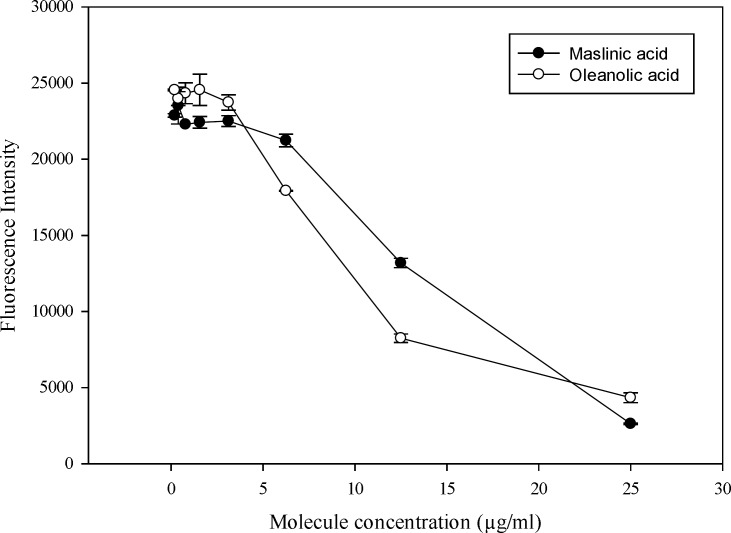
Cytotoxicity effect of triterpenic acid on J774 cell line

**Table 1:** Anti-amastigote activity, cytotoxicity and selectivity index (SI) of the triterpenic acids oleanolic and maslinic acids

	**LC**_50 _ ** (µg/mL)**	***L.infantum***		***L.amazonensis***	
		IC_50_ Anti-amastigote (µg/mL)	SI	IC_50 _Anti-amastigote (µg/mL)	SI
Oleanolic acid	8.103 ± 0.177	0.999 ± 0.089	8.111	3.040 ± 0.505	2.665
Maslinic acid	13.327 ± 0.353	2.916 ± 0.066	4.570	1.417 ±0.401	9.405

LC_50_: Lethal Concentration that reduces 50% of the L774 cell viability (µg/mL)

IC_50_: Inhibitory Concentration that inhibits 50% of the growth of the intra-macrophagic Leishmania (µg/mL)

*Parasite strains*

The activity of both molecules was evaluated against the amastigote form of *L. infantum *(MHOM/ES/1996/BCN-143**)** and *L. amazonensis *(MHOM/BR/77/LTB0016)

*Anti-amastigote Activity using the Alamar blue*

The anti-amastigote activity was measured according to Jain *et al*. (2012) (5). Macrophages of the J774 cell line were placed in a 96-well flat bottom plate at a density of 3 × 10^5^/mL in RPMI supplemented with 10% SBF and was incubated overnight at 37 °C in a 5% CO_2_ environment to allow almost complete differentiation of the cells. 100 µL of stationary phase promastigotes (7 days old culture) were added in a 10:1 ratio and the cultures (3 × 10^6^/mL) and were re-incubated at 37 °C for 24 h. to allow the parasites to infect the differentiated cells. After the incubation, the excess of promastigotes was washed off with the same medium at least 3 times. 100 μL of the culture medium (RPMI-1640 with 10% FBS) were added into each well. In the same time, a serially dilution of the test compounds was made in a 96-deep well plate with the same medium, and then 100 L of this serially-diluted standard were added to each well. The plates were incubated at 37 °C, 5% CO_2_ for 24 h. After this incubation, we remove the medium from each well and add 30 μL of RPMI-1640 for *L.infantum* and Schneider for *L.amazonensis* (with 0.05% SDS) was added to each well. Shake the plate for 30 sec and add 180 μL complete RPMI-1640 (with 10% FBS) or Schneider to each well. Incubate the plates at 26 °C for 48 h. for transformation of rescued amastigote to promastigotes. Add Alamar Blue at 10% into each well of the 96-well plates and incubate them at 26 °C for overnight. After overnight incubation, the plates were read in a spectrofluorimeter at 544 nm excitation, 590 nm emission.

*Cytotoxicity against mammalian cells*

Cytotoxicity was evaluated after 24 h. incubation of macrophage, the J 774 cell line, with different concentration of the tested molecules. The viability of the macrophages was determined with the Alamar Blue assay. Dose response curves were plotted and the CC_50 _were obtained. The analyses were performed in triplicate. 

## Results and Discussion

In a previous paper, we studied the leishmanicidal activity of olive leaf extract on the promastigotes stage of *Leishmania infantum* and *Leishamnia amazonensis *(4). 

The bio-guided fraction led to the isolation of two triterpenic acids oleanolic and maslinc acids. In the present study, the *in vitro* activity against amastigote form of Leishmania (L.) infantum and Leishmania (L.) *amazonensis *was investigated. Figure 1 shows that the isolated molecules from olive leaf extract, exhibited a dose dependent cytotoxicity against macrophage J 774. Meanwhile, within an amount of 5 µg/mL both molecules did not affect the cell development.

As observed in Table 1, the Maslinic acid showed the highest activity, against *L. amazonensis, *with an IC_50_ of 1.417 ± 0.401 µg/mL and a selectivity index of 9.405. Meanwhile, the oleanolic acid exhibit a higher activity against *L. infantum *with an IC_50_ of 0.999 µg/mL ± 0.089 and selectivity index of 8.111.

Similar results have been reported by Passero *et* *al*., (2011) (6), in their study, it was observed that the mix of oleanolic and ursolic acids was very potent against the amastigote form of *L.amazonensis *with an IC_50_ of 0.020 ± 0.007 µg/mL. In the same field, Torres-Santos *et al.* (2004) shows that the oleanolic acid could inhibit the amastigote form of *L amazonensis* with an IC_50_ about 11 µg/mL (7). 

Yamamoto *et* *al*., (2014) have studied, *in vivo*, the effect of triterpenic fraction purified from *Baccharis uncinella* leaves on the *in- vivo* inhibition of *Leishmania* *amazonensis, *the authors demonstrated that the fraction containing both oleanolic and ursolic acids inhibited the development of skin lesions without affecting the human cells (8). 

## Conclusion

Despite the fact that both *Leishmania *species tested in this study are able to cause different forms of leishmaniasis, both molecules tested seems to possess selectivity leishmanicidal properties against both strains. 
